# The A Allele of the Single-Nucleotide Polymorphism rs630923 Creates a Binding Site for MEF2C Resulting in Reduced CXCR5 Promoter Activity in B-Cell Lymphoblastic Cell Lines

**DOI:** 10.3389/fimmu.2016.00515

**Published:** 2016-11-17

**Authors:** Nikita A. Mitkin, Alisa M. Muratova, Anton M. Schwartz, Dmitry V. Kuprash

**Affiliations:** ^1^Laboratory of Intracellular Signaling in Health and Disease, Engelhardt Institute of Molecular Biology, Russian Academy of Sciences, Moscow, Russia; ^2^Department of Immunology, Lomonosov Moscow State University, Moscow, Russia

**Keywords:** CXCR5, multiple sclerosis, single-nucleotide polymorphism, rs630923, B-cells

## Abstract

Chemokine receptor CXCR5 is highly expressed in B-cells and under normal conditions is involved in their migration to specific areas of secondary lymphoid organs. B-cells are known to play an important role in various autoimmune diseases including multiple sclerosis (MS) where areas of demyelinating lesions attract B-cells by overexpressing CXCL13, the CXCR5 ligand. In this study, we aimed to determine the functional significance of single-nucleotide polymorphism rs630923 (A/C), which is located in *cxcr5* gene promoter, and its common allele is associated with increased risk of MS. Using bioinformatics and pull-down assay in B-lymphoblastic cell lines, we showed that protective minor rs630923 “A” allele created functional binding site for MEF2C transcription factor. Elevated MEF2C expression in B-cells correlated with reduced activity of *cxcr5* promoter containing rs630923 “A” allele. This effect that was fully neutralized by MEF2C-directed siRNA may mechanistically explain the protective role of the rs630923 minor allele in MS. Using site-directed mutagenesis of the *cxcr5* gene promoter, we were unable to find any experimental evidence for the previously proposed role of NFκB transcription factors in rs630923-mediated CXCR5 promoter regulation. Thus, our results identify MEF2C as a possible mediator of protective function of the rs630923 “A” allele in MS.

## Introduction

Multiple sclerosis (MS) is an inflammatory and neurodegenerative disease, which affects human central nervous system (CNS) causing demyelination and axon loss (1). Initiation and progression of MS are associated with increased migration of autoreactive lymphocytes across blood-brain barrier (2). For a long time, T-lymphocytes have been considered as main mediators of inflammation and autoimmune response against CNS myelin antigens (3). This assumption was prompted by a number of observations such as discovery of activated T lymphocytes in MS plaques or stimulation of experimental autoimmune encephalomyelitis (EAE), a mouse model of MS, by myelin-reactive T-cells. Although increased concentrations of antibodies and activated complement products are typical for MS lesions, the role of B-cells in MS propagation remained unclear until recently (4). Modern concept of MS recognizes fundamental contribution of B-cells in its development. Cortical axon damage and demyelination in secondary MS patients are associated with formation of B-cell follicle-like structures in the cerebral meninges (5). The presence of B-lymphocytes and plasmablasts in cerebrospinal fluid and of plasma cells in brain tissues correlates with production of anti-myelin autoantibodies that induce acute inflammatory response (6, 7). B-cells in CNS can also function as antigen-presenting cells and provide costimulatory signals to autoreactive T-cells (8). Different populations of B-lymphocytes infiltrating CNS are able to produce proinflammatory and regulatory cytokines such as IL-6, LTα, TGF-β, and IL-10 (9). These B-cells also express CCL22 and CCLl7 chemokines that are known to attract Th2 type T cells that further stimulate B-cell differentiation and antibody production (10).

A significant role of genetic predisposition in MS pathogenesis is well established, with MS risk alleles identified for a range of immunologically relevant loci including HLA, IL7R, IL2RA, and CD40 (11). Recent genome-wide association studies (GWAS) produced dozens of additional loci associated with MS (12, 13). The majority of single-nucleotide polymorphisms (SNPs) associated with MS are located outside the protein coding regions, and many of these SNPs reside in promoter, enhancer, or non-coding transcribed regions of genes that are active in B-cells (14). These observations corroborate with the role of B-cells in MS progression and suggest that some of the MS-associated alleles may directly participate in MS-related gene regulation.

Single-nucleotide polymorphism rs630923 (A/C) is located in the area of chemokine receptor CXCR5 gene promoter and its common “C” allele was shown to be associated with increased risk of MS (15). Under physiological conditions, chemokine receptor CXCR5 is highly expressed by mature B-cells and is responsible for B-cell homing to secondary lymphoid organs (11). Chemokine CXCL13, the ligand of CXCR5, is overexpressed in areas of actively demyelinating lesions, and almost all B-cells infiltrates in the CNS demonstrate high CXCR5 expression (12, 16), suggesting that CXCR5–CXCL13 axis is the main driver of B-cell recruitment to areas of MS lesions. Here, we sought to determine whether the minor “A” allele, associated with MS, affects the regulation of CXCR5 expression in B-cells. Understanding the role that this minor allele plays in CXCR5 regulation in B cells might help to explain the importance of this SNP in MS. In this paper, we demonstrate that activity of the CXCR5 promoter containing the “A” allele appears to be controlled, at least in part, by the MEF2C transcription factor.

## Materials and Methods

### Cell Lines

Raji lymphoblastic cell line was kindly provided by Dr. S. E. Dmitriev (Belozersky Institute of Physico-Chemical Biology, Lomonosov Moscow State University). Daudi lymphoblastic cell line was obtained from Official Collection of Somatic Cell lines of D.I. Ivanovsky Institute of Virology of The Ministry of Health and Social Development of The Russian Federation. MCF-7 breast cancer cell line was kindly provided by Dr. E. Zabarovsky, Karolinska Institute (Stockholm, Sweden). MCF-7-2si cell line with suppressed p53 was described previously (17). Raji and Daudi cells were cultured in RPMI 1640 medium (Life Technologies, Carlsbad, CA, USA) supplemented with 10% fetal bovine serum. MCF-7 cells were cultured in DMEM (Life Technologies, Carlsbad, CA, USA) supplemented with 10% fetal bovine serum and 0.01% human insulin. For activation, 100 ng/ml lipopolysaccharide (LPS), 5 ng/ml PMA, and 20 ng/ml ionomycin were added to cell culture for 24 h.

### Ethical Approval

Scientific Council of the Engelhardt Institute of Molecular Biology declared no ethical approval requirements for experiments performed in this study, because only commercially available cell lines were used.

### Pull-Down Assay

We used PCR to amplify a 155-bp fragment of the CXCR5 promoter (−112/ + 43) containing rs630923. Using two-step PCR, we generated the variants of this fragment containing “A” allele of rs630923 and point mutation of MEF2C-binding site. An additional specificity control represented a DNA fragment from an upstream genome location, which did not contain any MEF2C-binding sites (Table 1). All PCR products were verified by sequencing; sequences of all PCR primers and other synthetic oligonucleotides are represented in Table 1. Nuclear extracts from Raji and Daudi cells were isolated according to the published protocol (18). Nuclear extracts were incubated with DNA fragments and anti-MEF2C antibodies (ab79436, Abcam, Cambridge, UK) and precipitated with pre-blocked protein A sepharose beads. We quantified target DNA by real-time PCR after protein–DNA complexes elution and DNA purification. Background concentrations of the target DNA obtained for control reactions without lysate and without antibodies were subtracted from the data, which was subsequently normalized to the amplicon with mutant MEF2C-binding site. Rabbit IgG antibodies of irrelevant specificity representing an isotype control to the MEF2C antibody produced similar results for all DNA fragments regardless of the presence of the MEF2C-binding site.

**Table 1:** **Oligonucleotides used in the work**.

Oligonucleotide name	Sequence
**Primers for Real-time PCR**	
β-actin fw	5′-TGCGTGACATTAAGGAGAAG
β-actin rev	5′-GTCAGGCAGCTCGTAGCTCT
CXCR5 fw	5′-GCTAACGCTGGAAATGGA
CXCR5 rev	5′-GCAGGGCAGAGATGATTT
Mef2c fw	5′-TGGCAACAGCAACACCTACA
Mef2c rev	5′-ATCTCGAAGGGGTGGTGGTA
**Primers for amplification of CXCR5 promoter, point mutagenesis and amplification of fragments for pull-down assay**	
Prom fw	GGTAAGCTTCTCGGCCTTCCAAAGTTGATT
Prom rev	TAACCATGGAGAATTCCAGACAGGGCCTC
SNPreplace fw	GTGAGGGCAGCCTTTAAAATAGTCATAGGC
SNPreplace rev	ACTGGGGCCTATGACTATTTTAAAGGCTGC
Mef2cMut fw	GCAGCCTTTAAAATAGTCATAG
Mef2cMut rev	CTATGACTATTTTAAAGGCTGC
Pull-down fw	TGACTTTGCGTGGTGGTTTC
Pull-down rev	CACAGCATCAGTGCTAGTCAA
Pull-down control fw	TGATCTCGAACTCCTGACCT
Pull-down control rev	GTGCTGTGATTACAGGTGTG
**Primers for site-directed mutagenesis of NFkB sites**	
mut1 fw	GACTTAGCGGTTTCTGCAGTCAAGGGACT
mut1 rev	TTGACTGCAGAAACCGCTAAGTCCTGGCA
mut2 fw	TGATCAGCAGTTGAGCTCATCAACCTGCT
mut2 rev	GAGCTCAACTGCTGATCAACAGAGAACAG
mut3 fw	CTGTGGGCGATTTGAGCTCTTTCTTCAAA
mut3 rev	AGAAAGAGCTCAAATCGCCCACAGCATCA
**siRNAs for Mef2C knockdown**	
1-sense	CAACAUAUGGUACCGAGUAUU
1-antisense	UACUCGGUACCAUAUGUUGUU
2-sense	GAAUAACCGUAAACCAGAUUU
2-antisense	AUCUGGUUUACGGUUAUUCAU
Scrambled-sense	GACGUAACGCUUAGUAACAUU
Scrambled-antisense	UGUUACUAAGCGUUACGUCUU

### RNA Isolation, Reverse Transcription, and Real-Time Quantitative RT-PCR

Total RNA was extracted from cells using Trizol reagent (Invitrogen, Carlsbad, CA, USA) according to the manufacturer’s protocol. cDNA was generated using M-MULV reverse transcriptase and oligo-dT primer from First strand cDNA synthesis kit (Thermo Scientific, Waltham, MA, USA). Quantitative RT-PCR was performed using Applied Biosystems 7500 real-time PCR amplifier, Real-time PCR reaction mix containing Rox and SYBR Green (Evrogen, Moscow, Russia) and specific primers designed to amplify intron-spanning fragments of human ACTB, CXCR5, and MEF2C genes (Table 1). The PCR program included preheating stage at 95°C for 10 min and 40 cycles of amplification at 95°C for 15 s, 62°C for 20 s, and 72°C for 20 s. Melting curve analysis was used to assess the specificity of amplification. ACTB mRNA coding for human β-actin was used for normalization.

### MEF2C Knockdown Using siRNA

Commercially synthesized single-stranded RNA (Syntol, Moscow, Russia) were annealed by mixing sense and antisense RNA and slow cooling down from 90°C to room temperature in annealing buffer (5×: 50 mM Tris, 100 mM NaCl, pH 8.0). Raji cells were electroporated with siRNA duplexes (500 pmol per 5 million cells) 24 h prior to electroporation with luciferase constructs at which time 200 pmol more of the same siRNA duplex was added to prolong the silencing effect. We used two previously published (19) pairs of siRNAs targeting MEF2C (see Table 1). The second siRNA pair that turned out to be more efficient was used in all subsequent experiments.

### Western Blot Analysis

Total cell lysates were prepared using 5× Laemmli buffer. Protein samples (2 million cells per sample) were resolved on 12% SDS-PAGE, transferred to Hybond-C Extra nitrocellulose membrane (Amersham Biosciences, Amersham, UK), stained with anti-MEF2C antibodies (ab79436, Abcam, Cambridge, UK) at 1:2000 dilution and with HRP-conjugated secondary anti-rabbit antibody at 1:30,000 dilution. The bands were visualized with ECL using SuperSignal West Dura Extended Duration Substrate (Thermo Scientific, Waltham, MA, USA). Ponceau staining served as a loading control. Gel images were analyzed using ImageJ software to determine the integral intensities of the ECL bands and of the lanes of Ponceau-stained blots.

### Reporter Constructs and Site-Directed Mutagenesis

We amplified the (−455/ + 368) region of human *cxcr5* promoter (17, 20) by PCR using genomic DNA from Raji cells as a template and specific primers containing cloning sites. CXCR5 promoter variants containing minor “A” variant of rs630923 or mutation in predicted MEF2C-binding site were generated by two-step PCR mutagenesis and verified by sequencing (see Table 1 for sequences of all oligonucleotides). All variants of CXCR5 promoter were cloned into pGL3-basic luciferase reporter construct (Promega, Madison, WI, USA) using *Hin*dIII and *Nco*I restriction sites.

### Electroporation and Luciferase Reporter Assay

Cells were electroporated with 5 μg of purified plasmid DNA, and 0.1 μg of pRL-CMV renilla luciferase control reporter vector (Promega, Madison, WI, USA) using Neon Transfection System (Thermo Scientific, Waltham, MA, USA). For each cell line, we used protocols provided by the manufacturer: one 30 ms 1300 V pulse for Raji, two 30 ms 1100 V pulses for MCF-7, and two 30 ms 1300 V pulses for Daudi. Luciferase activity was measured in Luminometer 20/20^n^ (TurnerBioSystems, Sunnyvale, CA, USA) using Dual-Luciferase Reporter Assay System (Promega, Madison, WI, USA) following the manufacturer’s protocol. The activity of Firefly luciferase was normalized to that of renilla luciferase to account for variations in transfection efficiency.

### Statistical Analysis

We used Microsoft Excel and Statistical software for statistical analyses. Statistical significance was determined using two-tailed unpaired Student’s *t*-test, *P*-value <0.05 was considered significant. Data for each sample represent the result of at least three independent experiments. Real-time PCR and luciferase assays were additionally performed in two technical replicates. Error bars in the graphs represent SEMs value.

## Results

### Minor MS Allele of SNP rs630923 in CXCR5 Promoter Creates MEF2C-Binding Site

Single-nucleotide polymorphism rs630923 (A/C) is located in a highly conservative area of chemokine receptor CXCR5 gene promoter [−31 with respect to the transcription start site (TSS)] and its common “C” allele was shown to be associated with increased risk of MS (12, 15). Since nucleotide variations this close to the TSS may affect the efficiency of transcriptional initiation, we hypothesized that rs630923 may influence CXCR5 promoter activity directly and searched the CXCR5 promoter region for transcription factor binding sites (TFBS) that either appear or disappear depending on the rs630923 allele. We used PERFECTOS-APE software (21) with curated TFBS models from JASPAR and HOCOMOCO databases. The list of candidate transcription factors with best scores included MEF2C, MEF2A, FoxL1, DUX4, HNF4G, and TCF7L2. Of these, MEF2C stood out as the most relevant candidate since it is highly expressed in B-cells (22), participates in inflammation-induced B-cell proliferation (23), and targets a subset of B-cell-specific genes (24). MEF2C was also shown to influence migration potential of leukemic cells by targeting CCR2 and CCR5 chemokine receptors genes (25). Since B-cells facilitate the progression of MS (26), we predicted that the minor “A” rs630923 allele may play its protective role by creating a binding site for MEF2C that attenuates the transcription of CXCR5 gene encoding the critical chemotactic receptor on these cells.

**Figure 1:**
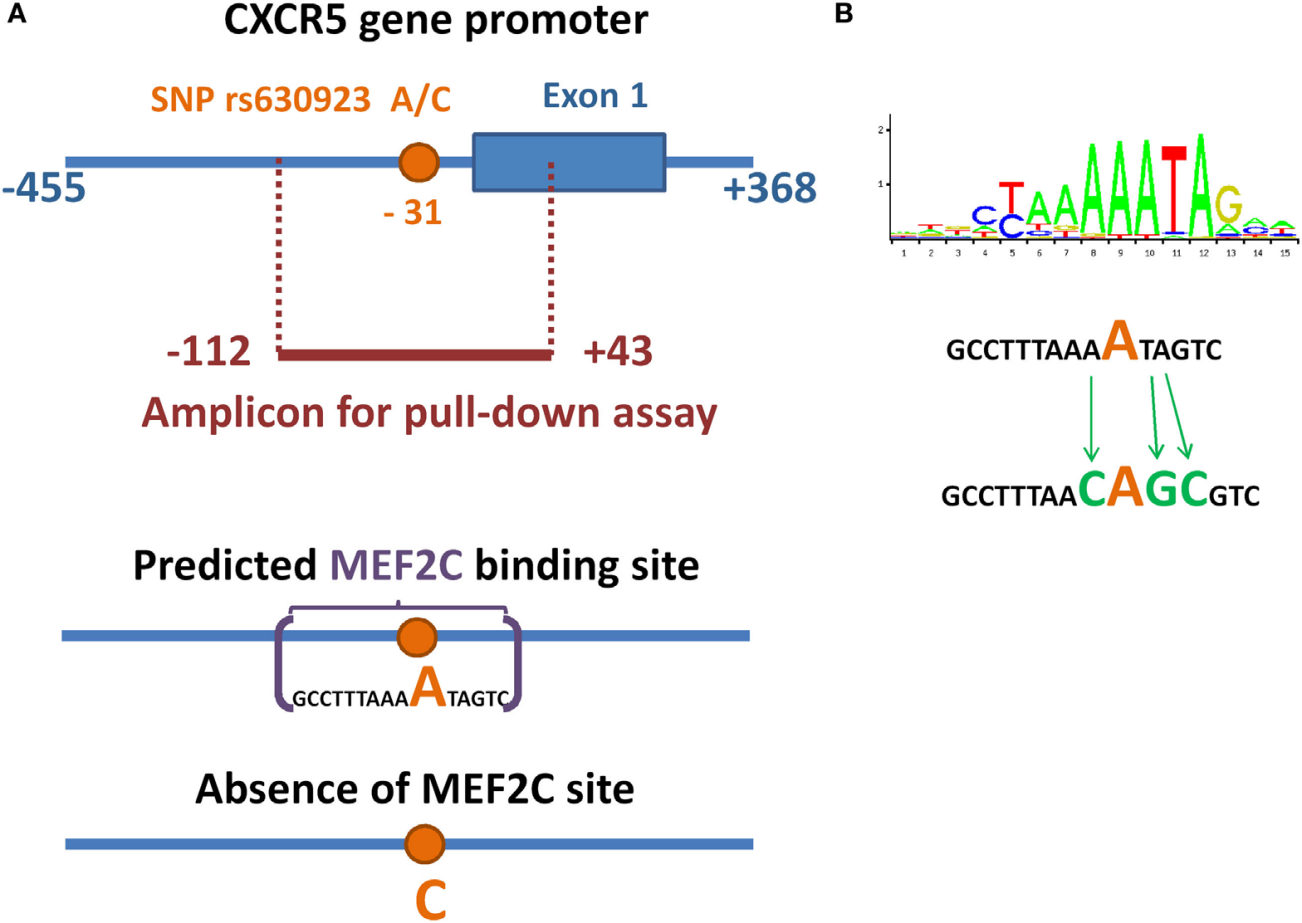
**Minor allele of SNP rs630923 creates a MEF2C-binding site**. **(A)** Schematic illustration of location of SNP rs630923 and MEF2C-binding site, associated with its “A” allele, in CXCR5 promoter. **(B)** Position weight matrix of MEF2C-binding site and the scheme of its point mutagenesis.

We used pull-down assay to investigate the influence of rs630923 on MEF2C binding to the CXCR5 promoter. We constructed three variants of CXCR5 gene promoter (−112/ + 43) region (Figure 1A) containing either common “C” rs630923 allele, minor “A” allele or a combination of the minor “A” allele with additional mutations disrupting the MEF2C consensus (Figure 1B). Pull-down assay was performed using nuclear extracts from two human lymphoblastic B cell lines, Raji and Daudi (both cell lines carry C/C rs630923 alleles). High levels of CXCR5 and MEF2C expression are typical for both cell lines, according to Broad-Novartis Cancer Cell Line Encyclopedia (27).

**Figure 2:**
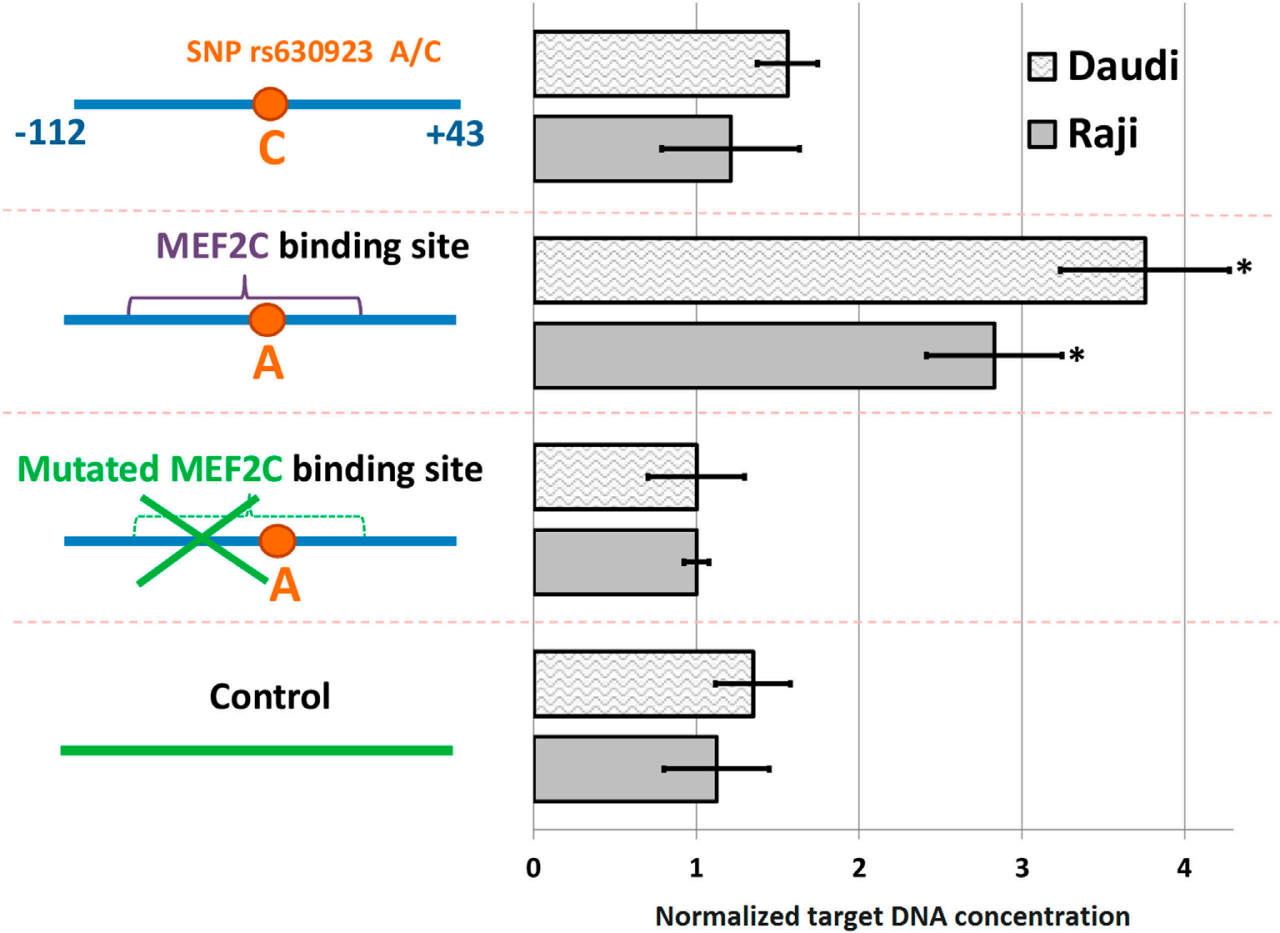
**MEF2C binding to CXCR5 promoter depends on the rs630923 allele**. The efficiency of MEF2C binding was estimated by pull-down assay using nuclear extracts from Raji and Daudi B-lymphoblastic cell lines. DNA fragments used for binding are schematically depicted on the left, with variants of the CXCR5 promoter fragment as blue line segments and control fragment of CXCR5 locus as green line segment. Data were normalized to the amplicon with mutant MEF2C-binding site after subtraction of the background values (concentrations of the target DNA obtained for control reactions without lysate and without antibodies). The result of three independent experiments is shown. *P < 0.05.

A significant amount of the DNA probe containing minor “A” rs630923 allele could be precipitated from the binding reaction with nuclear extracts from both cell lines using anti-MEF2C antibodies while the “C” probe did not significantly differ from the control probes, including the one with point mutation of the MEF2C site (Figure 2). These data demonstrate the presence of a MEF2C-binding site in the CXCR5 promoter containing minor rs630923 allele.

### Presence of Active MEF2C-Binding Site Is Associated with Reduced CXCR5 Promoter Activity

**Figure 3:**
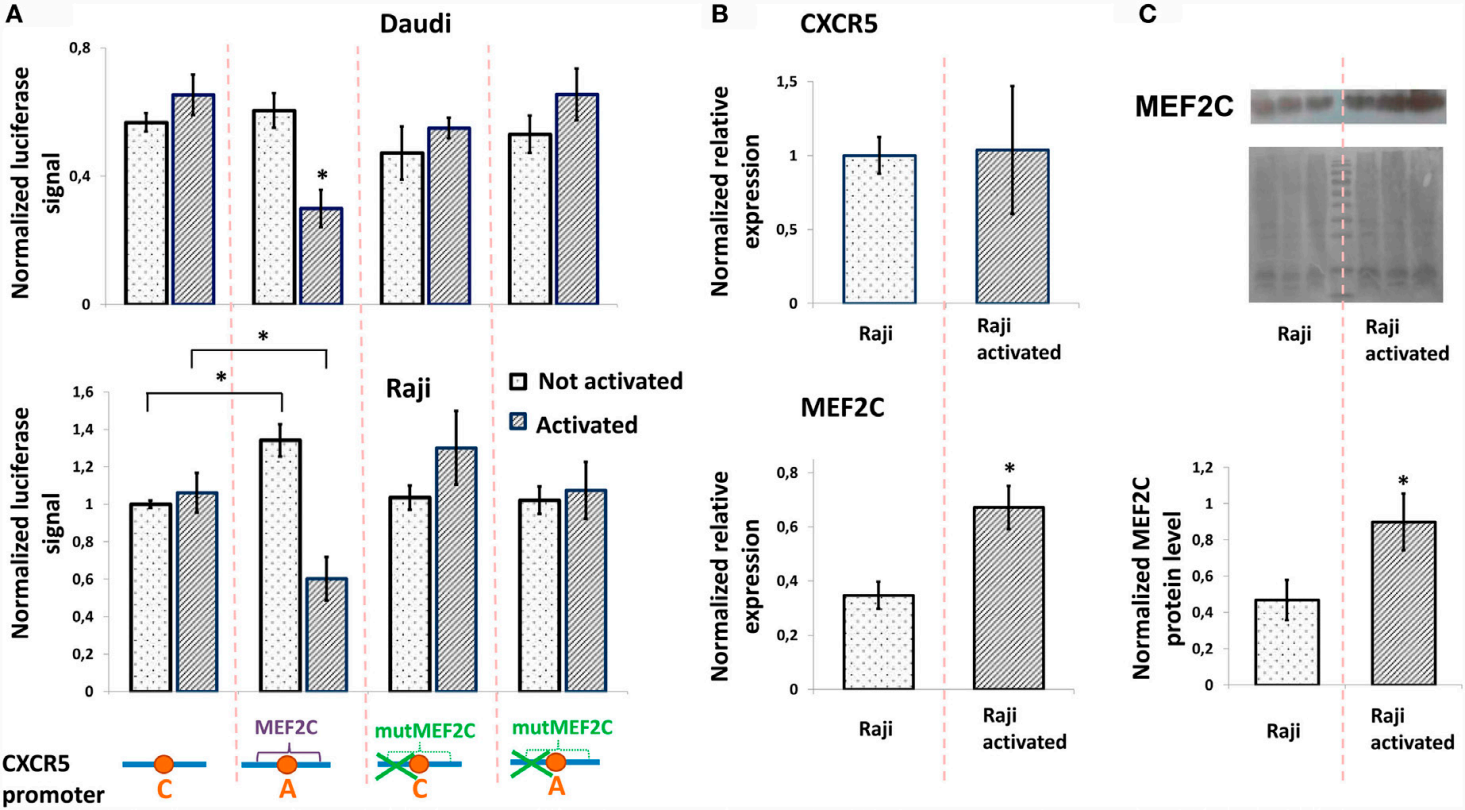
**Minor “A” allele of rs630923 is responsible for reduced CXCR5 promoter activity in activated B-lymphoblastic cells**. **(A)** Minor “A” allele of rs630923 reduces the CXCR5 promoter activity specifically in activated B-cells. The data shown were obtained in five independent experiments and normalized to renilla luciferase activity. *P < 0.01. **(B)** Activation does not influence the level of CXCR5 expression (top panel) but leads to increased MEF2C mRNA (bottom panel). The real-time RT PCR data shown are the result of three replicate experiments. **(C)** MEF2C protein is increased in activated Raji cells. A representative western blot is shown (top panel). The experiment was repeated three times, gel images were quantified using ImageJ software (bottom panel). *P < 0.05.

To study the role of rs630923 and associated MEF2C-binding site in modulation of CXCR5 promoter activity, we generated luciferase reporter constructs containing CXCR5 promoter variants both rs630923 alleles with or without the MEF2C-binding site mutation (Figure 3A). In order to emulate BCR stimulation and costimulatory signals received by B cells migrating to MS lesions (28), we stimulated Raji and Daudi B-lymphoblastic cells using combination of LPS, PMA, and ionomycin. B-cell activation leads to increased MEF2C expression (22) but does not influence CXCR5 expression level (29, 30). These previously published observations were verified for CXCR5 by real-time RT-PCR using mRNA from Raji cells (Figure 3B) and for MEF2C by RT-PCR (Figure 3B) and by western blot (Figure 3C).

The presence of either rs630923 “A” allele and/or MEF2C-binding site did not significantly influence the CXCR5 promoter under normal culture conditions, with the exception of the activity of the rs630923 “A” allele variant in Raji cells that was somewhat elevated in this particular set of experiments. However, B-cell activation led to a significant and specific decrease in CXCR5 promoter activity in both B-cell lines when the promoter contained the minor “A” rs630923 variant in the context of a functional MEF2C-binding site (Figure 3A). These data indicate that protective role of “A” rs630923 allele in MS (presumably associated with low CXCR5 and reduced migration of activated B-cells to areas of demyelinating lesions) may be explained by repression of CXCR5 promoter activity by MEF2C transcription factor.

**Figure 4:**
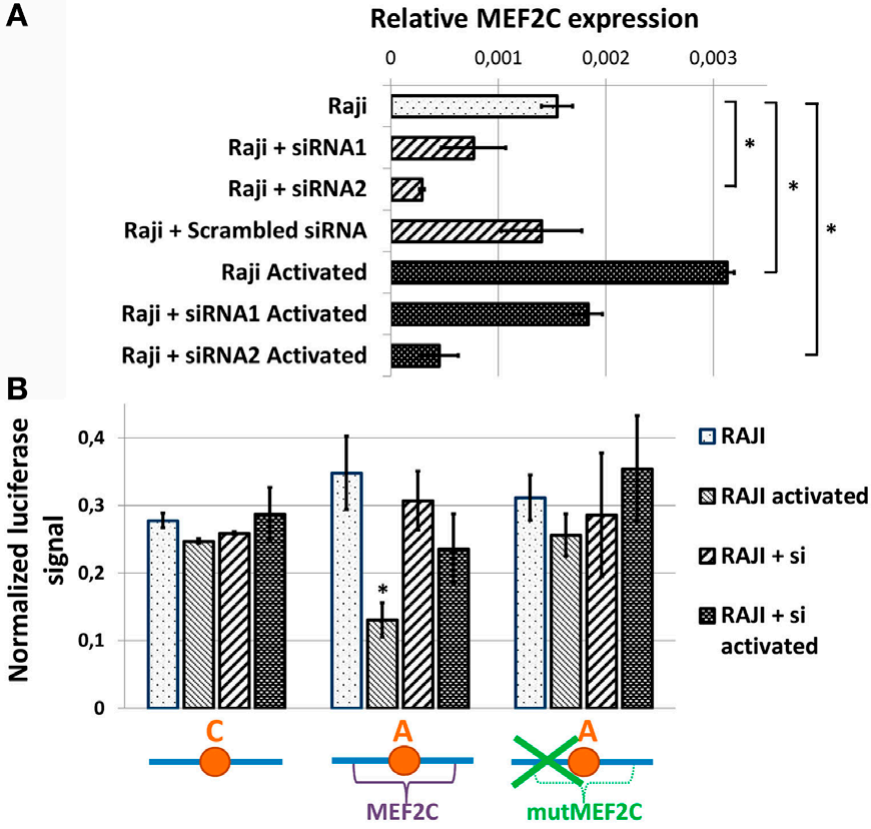
**Reduced activity of CXCR5 promoter with minor “A” rs630923 variant in activated B- cells depends on MEF2C expression**. **(A)** MEF2C expression in Raji cells electroporated with two different variants of anti-MEF2C siRNAs and control scrambled siRNA before and after B-cell activation. MEF2C mRNA was measured by real-time RT-PCR and normalized to ACTB (β-actin). The result of three independent experiments is shown. **(B)** The decrease in CXCR5 promoter activity in activated Raji cells associated with minor “A” rs630923 allele is completely abolished by MEF2C knockdown. The data shown were obtained in five independent experiments and normalized to renilla luciferase activity. *P < 0.05.

To assess the role of MEF2C transcription factor directly, we evaluated the activities of various CXCR5 promoter variants in Raji cells upon siRNA-mediated suppression of MEF2C expression. We tested two variants of previously published siRNAs against MEF2C (see Materials and Methods), and one of them that caused up to 80% decrease in MEF2C mRNA level and insensitivity of MEF2C expression to cell activation (Figure 4A) was used in subsequent experiments. The activity of CXCR5 promoter lacking MEF2C-binding site (either “C” allele or “A” allele with MEF2C-binding site mutation) was essentially insensitive to both cell activation and MEF2C expression level. However, MEF2C knockdown completely abolished the decrease in the activity of CXCR5 promoter containing the protective “A” allele that was observed in activated cells (Figure 4B). These data indicate that in our cell culture model, both MEF2C and rs630923 protective alleles play unique and essential roles in the reduction of CXCR5 promoter activity during B-cell activation.

### NFkB Status Does Not Influence the rs630923-Dependent CXCR5 Promoter Activity in MCF-7 Breast Cancer Cells

**Figure 5:**
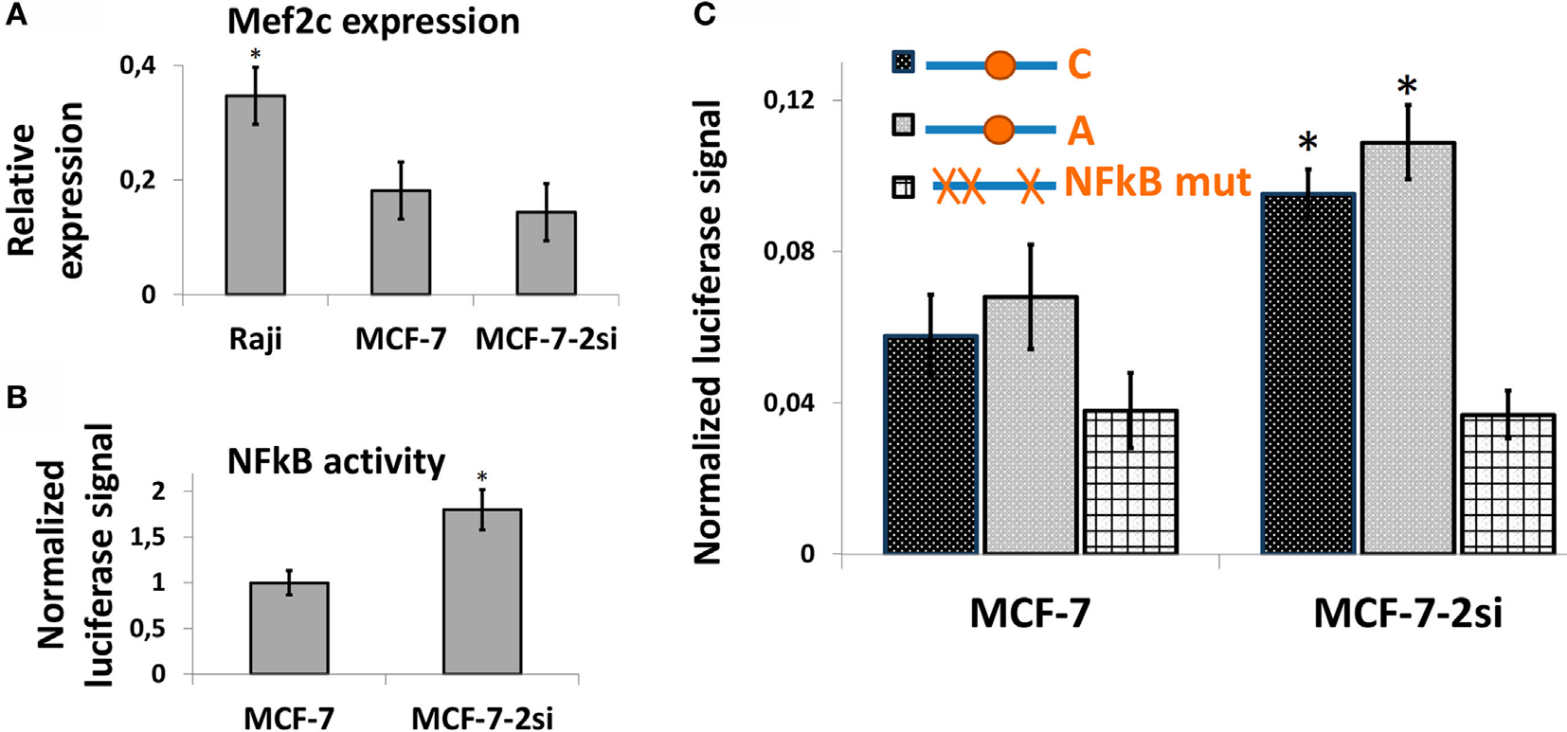
**The effect of rs630923 SNP on CXCR5 promoter activity in MCF-7 breast cancer cells is not sensitive to NFkB status**. **(A)** The level of MEF2C mRNA in MCF-7 breast cancer cells is only two times lower than in Raji cells and is not significantly sensitive to p53 knockdown. **(B)** NFkB activity is significantly higher in MCF-7 cells with p53 knockdown. **(C)** CXCR5 promoter activity in MCF-7 cells is sensitive to NFkB status but not to the rs630923 SNP. The data shown were obtained in five independent experiments and normalized to renilla luciferase activity. *P < 0.05.

It has been suggested earlier that an NFkB binding site in the vicinity of rs630923 may play a role in cxcr5 promoter regulation (15), whereas our own studies of CXCR5 promoter did not reveal any NFkB-binding sites overlapping this SNP (17). To directly access the role of rs630923 in NFkB-dependent CXCR5 promoter activity we used MCF-7 breast cancer cell line and its derivative cell line MCF-7-2si with p53 knockdown (17). This cell culture model takes advantage of the functional antagonism between p53 and NFκB (31) that results in elevated NFκB activity in the absence of p53-mediated suppression (17). Both cell lines showed significant MEF2C expression (Figure 5A). This observation correlated with publically available expression data (27) and indicated that MEF2C effects on CXCR5 promoter could be addressed in this system. The level of NFkB activity, as assessed by an NFkB-dependent reporter plasmid, was significantly higher in MCF-7-2si cells (Figure 5B), again in agreement with our previous results (17). We then measured the activities of the CXCR5 promoter versions containing “C” and “A” alleles of the rs630923 SNP using previously described promoter variant with mutations in all NFkB-binding sites as a negative control (Figure 5C). Both test constructs were significantly more active in MCF-7-2si cells as compared to the parental MCF-7 cell line, but no significant difference between two allelic variants was observed. This result indicates that although CXCR5 promoter activity is modulated by NFkB, rs630923 is not involved in this process.

## Discussion

Modern concept of MS pathogenesis implies an important role for B-cells in disease progression (4). B-cell migration to areas of demyelination depends on interaction of CXCL13 chemokine with CXCR5 chemokine receptor (16).

In this work we report functional characterization of the SNP rs630923 (A/C) located 31 bp upstream of the TSS of the CXCR5 gene. Using bioinformatics, we identified MEF2C transcription factor that is known to enhance B-cell proliferation and survival in response to BCR stimulation (22, 23) as the most likely candidate for molecular discriminator between the rs630923 alleles. Pull-down DNA binding assay with nuclear extracts from activated B-cells confirmed the prediction and demonstrated MEF2C binding to the minor “A” but not to the common “C” rs630923 variant (Figure 2). CXCR5 promoter containing the “A” rs630923 variant demonstrated reduced ability to drive reporter gene transcription in activated B-cells (Figure 3); importantly, the effect was MEF2C-dependent (Figure 4). MEF2C has been described as an activator as it induces expression of a number of chemokines and their receptors (24), and MEF2C loss is associated with poor homing of leukemic cells (25). However, in this system, MEF2C appears to act as a repressor of CXCR5 gene transcription in activated B-cells. B-cell activation has been implicated in reduced CXCL13-directed B-cell migration (32). Proposed mechanism of this process focused on the downstream stages of the CXCL13/CXCR5-signaling cascade, taking into account known CXCR5 insensibility to B-cell activation (29). Our data indicate that in an appropriate genomic context, direct regulation of the CXCR5 gene may be involved as well.

Another question regarding CXCR5 expression in MS was previously proposed NFkB involvement in rs630923-mediated CXCR5 promoter regulation (15). Our previous experiments on CXCR5 promoter regulation in an NFkB-dependent system based on breast cancer cell lines (17) did not reveal any functional NFkB sites in the area of interest. We now directly tested the activities of CXCR5 promoter variants with different rs630923 alleles in the same system and did not find any evidence of rs630923 involvement in NF-B-dependent CXCR5 gene regulation (Figure 5).

In conclusion, our data suggest that protective role of the minor variant of rs630923 SNP in MS may result from intricate interplay between B-cell activation and migration mechanisms. In the homozygous carriers of the protective allele, BCR activation that is typical for MS would be counteracted by MEF2C-dependent downregulation of CXCR5 gene expression. That would result in a lower rate of CXCL13-directed B-cell migration to secondary lymphoid organs, reduced CNS infiltration and reduced rate of meningeal B-cell follicles formation (33), providing a possible mechanistic explanation for the low risk of MS progression associated with the rs630923 minor allele. Whether this mechanism actually affects B-cell migration in MS patients should be verified in future studies.

## Author Contributions

NM carried out most of the experiments, analyzed the data, designed the study, and drafted the manuscript. AM participated in real-time PCR analysis and promoter studies. AS participated in study design and bioinformatic analysis. DK supervised the study, analyzed the data, and revised the manuscript.

## Conflict of Interest Statement

The authors declare that the research was conducted in the absence of any commercial or financial relationships that could be construed as a potential conflict of interest.
